# Vertical root fracture resistance and crack formation of root canal-treated teeth restored with different post-luting systems

**DOI:** 10.1007/s10266-022-00709-5

**Published:** 2022-05-06

**Authors:** Andreas Rathke, Henry Frehse, Beatrice Hrusa

**Affiliations:** 1 Faculty of Medicine, University of Ulm, Albert-Einstein-Allee 7, 89081 Ulm, Germany; 2 Dentsply Sirona, Konstanz, Germany; 3 Mutlangen, Germany

**Keywords:** Dentin crack, Fiber-reinforced composite, Post, Staircase, Vertical root fracture

## Abstract

The aim was to investigate the vertical root fracture (VRF) resistance and crack formation of root canal-treated teeth restored with different post-luting systems. Human maxillary lateral incisors of similar size were decoronated, assigned to five groups (*n* = 18, power = 0.9) and embedded in acrylic blocks with artificial periodontal ligament. After root canal filling, post spaces were prepared to place coated fiber-reinforced composite (FRC) or sandblasted titanium (Ti) posts of the same shape and size. Half of the posts were zinc phosphate cemented (C), while the other half was adhesively luted (A). Untreated teeth served as control. After thermal cycling and staircase loading in a chewing simulator, the crack formation on the root dentin surface was microscopically examined and classified as no defect, craze line, vertical crack, and horizontal crack. Subsequently, the samples were loaded until root fracture. Data were analyzed by one-way ANOVA, Tukey’s test, and Fisher’s exact test. All samples survived the chewing simulation without VRF, but crack formation was significantly different between the groups (*P* = 0.009). The control showed significantly fewer defects than FRC/C, Ti/C, and Ti/A (*P* = 0.001, *P* = 0.008, *P* = 0.008, respectively). FRC/C showed the highest incidence of vertical cracks. FRC/A had the lowest incidence of defects. There was no significant difference in VRF resistance between the groups (*P* = 0.265). Adhesively luted FRC posts did not increase VRF resistance but reduced the risk of defects. Most defects were craze lines and vertical root cracks.

## Introduction

Vertical root fractures (VRF) are serious adverse events that often result in the extraction of the tooth [1]. Although a low prevalence of VRF is reported in the literature [2], other investigations showed a risk of up to 32% [1, 3]. The VRF prevalence was significantly higher when a post was placed than in root canal-treated teeth without post [4]. Root canal treatment and post space preparation can damage the root dentin and lead to incomplete cracks or craze lines that can develop into VRF [5–7]. Research agrees that root canal-treated teeth cannot be reinforced with cemented metal posts, while adhesively luted fiber-reinforced composite (FRC) posts are a promising technique for reinforcing teeth [8–12]. FRC posts have a superior stress distribution due to their dentin-like Young’s modulus and the fact that they can be adhesively luted into the post space [8, 13]. Their potential to reduce the incidence of irreparable root fractures [8, 13] and to result in higher survival rates than metal posts was highlighted [14]. However, other studies found that FRC posts do not reinforce teeth [15, 16] and that both the prevalence of irreparable root fracture and the survival rate are comparable to that of metal posts [17]. Recent long-term clinical results showed a higher survival rate for adhesively luted titanium (Ti) posts than for adhesively luted FRC posts after up to 15 years [18, 19]. However, the high drop-out rate of over 40% and the low statistical power made the interpretation of the results susceptible to bias and did not allow sufficient evidence for the selection of FRC versus Ti posts [20]. Since the results are inconclusive so far, the most suitable post concept remains controversial.

Three-dimensional finite element method (3D FEM) analyses indicated that the favorable stress distribution of FRC posts can lose its impact over time if the adhesion to the root dentin fails and higher stress are concentrated in the root [21, 22]. Cracks were initiated when the concentrated stress exceeded the tensile strength of the root dentin. It has been demonstrated that cracks propagate under repeating subcritical loads and crack propagation increases the likelihood for future fractures [23]. However, studies usually addressed the fracture resistance and location of the fracture under static loading [13, 24]. Some authors focused on post-restored teeth under fatigue loading [7, 11, 16, 25]. One reason is that the number of cycles until root fracture is very high under physiological chewing loads [1]. Alternatively, staircase loading with gradually increasing load for a certain number of cycles was used for fatigue testing of post-restored teeth [11]. The aim of this study was to evaluate the influence of cemented and adhesively luted FRC or Ti posts on resistance against VRF and dentin defects after chewing simulation. The first null hypothesis to be tested was that the VRF resistance is not significantly different between the groups. The second null hypothesis was that there is no significant difference in the incidence of defects between the groups.

## Materials and methods

### Sample size calculation

The sample size was calculated using unpublished pilot data (*n* = 5) and the two-sided Welch’s t-test for unequal variance at a significance level of *P* ≤ 0.05 and a power of 0.9 (nQuery Advisor version 7, Statistical Solutions, Cork, Ireland). The sample size was evaluated as *n* = 15 for each group. Considering some dropouts and a deviation of normality assumptions a sample size of *n* = 18 was used in the study.

### Sample preparation

Extracted human maxillary lateral incisors with comparable dimensions were disinfected in accordance with the university’s policy. The teeth were cleaned with scalers and stored in 1% chloramine trihydrate solution. Crowns were removed using a diamond saw at slow speed (WOCO 50/Med, Conrad, Clausthal-Zellerfeld, Germany) to obtain roots of 13 mm length. A stereomicroscope (Stemi SV8, Zeiss, Oberkochen, Germany) at 12 × magnification was used to exclude any pre-existing dentin defects. After numbering the teeth, the cross sections of the roots were measured at the level of the cutting surface in the mesio-distal and bucco-palatal direction with a digital caliper (Garant, Hoffmann, Munich, Germany). The area of the ellipsed root cross-section (*A*) was calculated according to: *A* = π/4 × *a* × *b* (where *a* and *b* were the mesio-distal and bucco-palatal dimension in mm). Roots of extreme size were excluded. The remaining samples were randomly distributed into five groups of 18 roots each according to a random numbers table. To simulate the periodontal ligament with relatively uniform stress distribution, the roots were wrapped in one layer of latex rubber milk (Suter Kunststoffe, Jegenstorf, Switzerland) with a thickness of approximately 250 µm and embedded in acrylic resin (Technovit 4071, Heraeus Kulzer, Hanau, Germany) with the cervical root third being exposed.

In the control group, the roots were left untreated. In the other groups, the canals were instrumented using single-length technique with nickel-titanium rotary files (EasyShape, Brasseler Komet, Lemgo, Germany) up to file size 40 and 0.04 taper. The working length was set to 12 mm. During instrumentation, canals were irrigated with 5 ml of 3% sodium hypochlorite (NaOCl) and 15% ethylenediaminetetraacetic acid (EDTA) solutions (Glyde File Prep, Dentsply Sirona, Ballaigues, Switzerland). After a flush with 5 ml distilled water, the canals were dried with paper points and obturated with the matched EasyShape gutta percha cone and EasySeal resin-based sealer (Brasseler Komet) using the single-cone technique.

### Post placement

Drills supplied by the post manufacturer (Brasseler Komet) were used to prepare post spaces of 8 mm depth and size ISO 70. Gutta percha was removed with the pilot drill 183LB leaving 4 mm of the root filling in the apical portion. The root canals were enlarged with the reamer 196 and roughened with the form congruent diamond instrument 196D. After a flush with 5 ml distilled water, the post spaces were dried with paper points. Prefabricated coated FRC posts (ER DentinPost Coated) and sandblasted Ti posts (ER Kopfstift) in the same cylindroconical shape and size (ISO 70) were shortened from the coronal end to 7 mm with diamond drills under water cooling so that they could be placed into the post canals 1 mm below the cutting surface of each root. All posts were cleaned with 70% alcohol. For each post material, half of the samples were cemented (C), while the other half was adhesively luted (A). For cementation, zinc phosphate cement (Harvard, Richter & Hoffmann, Harvard Dental, Berlin, Germany) was mixed in a creamy consistency in relation 1.5 g zinc oxide powder to 1 g phosphoric acid on a cooled glass plate and applied to the post surface. After the post was cemented, the excess cement was removed. For adhesive luting, the dual-curing luting system of the posts (DentinBond/DentinBuild, Brasseler Komet) was applied according to the manufacturer’s instructions. Post spaces were etched with 37% phosphoric acid gel for 20 s, rinsed with water spray, and dried with paper points. The two-step etch-and-rinse adhesive DentinBond was applied in two consecutive coats using a microbrush. Excess adhesive inside the post space was removed with paper points. After solvent evaporation, the adhesive was light cured for 10 s. The dual-curing resin composite cement DentinBuild was applied to the post surface directly from the Minimix syringe provided by the manufacturer and the post was inserted into the post space. After excess removal, the resin cement was light cured for 20 s. Light-curing was performed with a halogen light-curing unit (Astralis 10, Ivoclar Vivadent, Schaan, Liechtenstein) at 1000 mW/cm^2^ light intensity. The cervical 1 mm of the post space was filled using a temporary filling material (Cavit, 3M Espe, Seefeld, Germany).

### Chewing simulation and VRF testing

After water storage for 24 h at 37 °C, the samples were subjected to 1500 thermocycles in distilled water at 5–55 °C with a dwelling time of 20 s in each bath (Haake W15, Willytec, Gräfelfing, Germany). Mechanical loading was performed according to the staircase method starting at a load of 25 N at an angle of 10° to the axial direction of the roots in a chewing simulator (Standard 2002, Willytec). Every 20,000 cycles at a frequency of 2 Hz, the load was increased in increments of 25 N until 120,000 cycles were reached. The unfilled cervical 1 mm of the post space ensured that the force applied by the cone-shaped metal antagonist with an angle of 90° was transmitted to the root dentin rather than the post. The diameter of the truncated cone was dimensioned in such a way that the cone tip fitted exactly into the unfilled cervical post space.

**Fig. 1:**
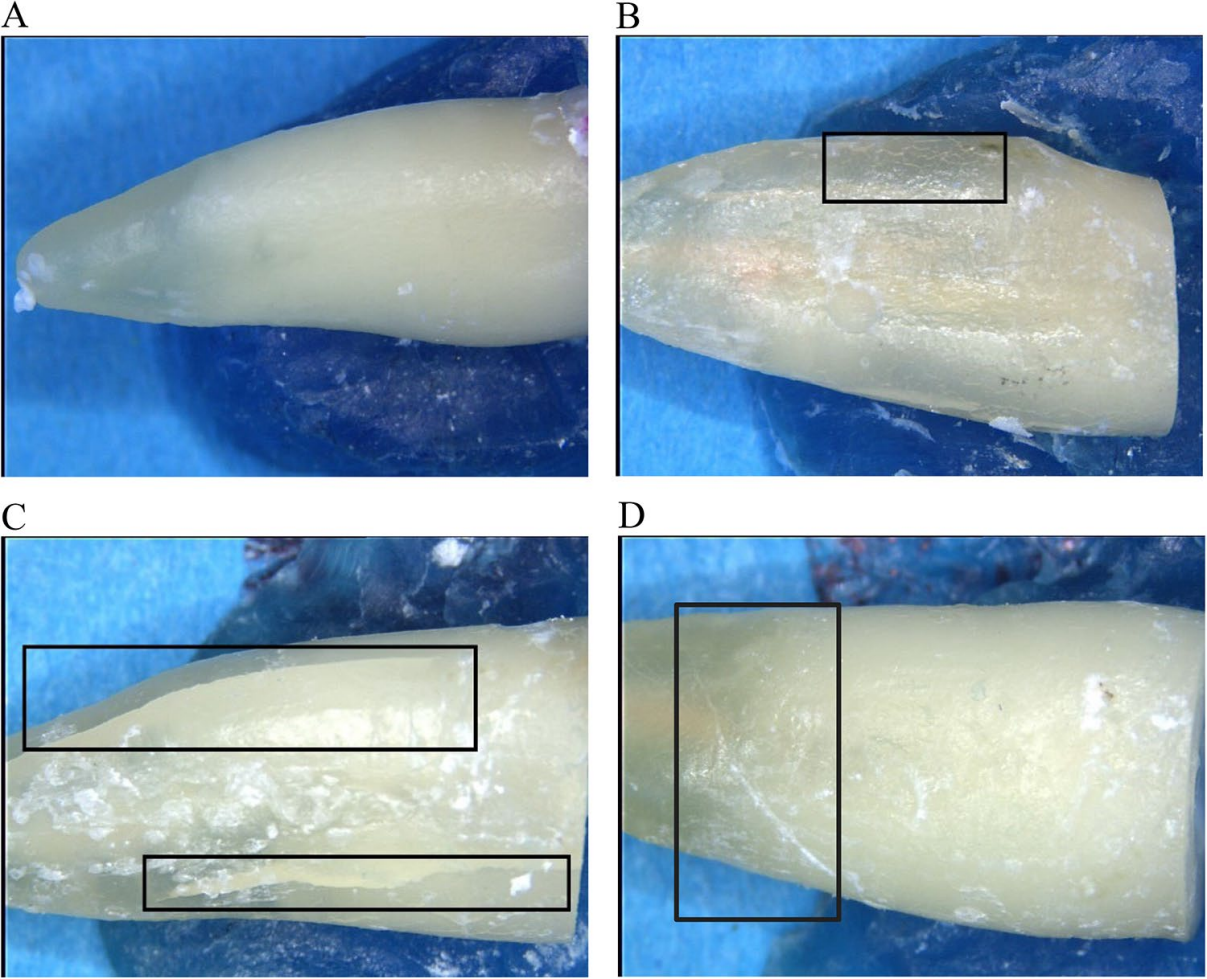
Representative microscopy images of the different dentin defects along the external root surface after chewing simulation. **A** No defect. **B** Craze line. **C** Vertical crack. **D** Horizontal crack. Original magnification 12×  in each case

After chewing simulation, the external root surfaces were examined under the microscope using a cold light source (Stemi SV8, Zeiss). Because of the latex milk, the roots could easily be removed from the acrylic blocks. Pictures were taken with a digital camera at 12–100 × magnification (3CCD Color Video Camera, Sony, Tokyo, Japan). Crack formation was analyzed per root third (cervical, middle, apical) as follows: (a) no defect, (b) craze line, (c) vertical crack, and (d) horizontal crack. Representative images of the dentin defects are shown in Fig. 1. After microscopic analysis, the roots were returned to the acrylic blocks and subjected to VRF testing. The same antagonist as used for the chewing simulator was attached to the load cell of a universal testing machine (Zwicki 1120, Zwick, Ulm, Germany). The samples were loaded until fracture with a crosshead speed of 1 mm/min. The fracture load (*N*) was recorded when the force in the load-strain curve decreased by 30%.

### Statistics

Statistical analysis was performed with IBM SPSS version 19 for Windows (Chicago, IL, USA). The significance level was set in advance at *P* ≤ 0.05. Differences in crack formation were evaluated by Fisher’s exact test. After confirming that the VRF resistance (*P* = 0.244) and the root cross-sectional area (*P* = 0.651) met the assumptions of normality as indicated by the Kolmogorov–Smirnov test, differences between the means of the groups were compared with one-way ANOVA followed by post hoc Tukey’s test.

## Results

The mean values for the VRF resistance, the mean cross-sectional area, and the crack formation are shown in Table 1. All samples survived the chewing simulation without a complete root fracture. The incidence of dentin defects was significantly different between the groups (*P* = 0.009). The control teeth showed significantly fewer defects than the post-luting groups FRC/C (*P* = 0.001), Ti/C (*P* = 0.008), and Ti/A (*P* = 0.008). FRC/C showed the highest incidence of defects, mainly vertical cracks. FRC/A showed the lowest incidence of defects that was not significantly different from that of the control teeth (*P* = 0.153). Among the post-luting groups, 44% of the samples showed dentin defects in the apical root third, while 66% and 72% of the samples had dentin defects in the cervical and middle root section, respectively.

**Table 1:** Mean and standard deviation of VRF resistance (*N*) and cross-sectional area (mm^2^) as well as the incidence of dentin defects (absolute number) of the control and post groups

Group	*N*	VRF resistance (N)	Area (mm^2^)	Dentin defect			
				No defect	Craze line	Vertical crack	Horizontal crack
Control	18	750.4 ± 214.3	20.46 ± 2.57	12	3	3	0
Ti/C	18	920.8 ± 327.7	20.95 ± 2.62	3	9	6	0
Ti/A	18	863.6 ± 311.2	22.19 ± 3.83	3	6	8	1
FRC/C	18	748.1 ± 238.8	20.35 ± 2.93	2	4	12	0
FRC/A	18	866.4 ± 304.6	20.54 ± 2.03	6	7	5	0
Total	90	829.9 ± 285.1	20.94 ± 2.89	26	29	34	1

No significant differences between the groups regarding VRF resistance (*P* = 0.265) and cross-sectional area (*P* = 0.402)

*A* adhesive luting; *C* cementation; *FRC* fiber-reinforced composite; *Ti* titanium; *VRF* vertical root fracture

No significant differences were found between the groups regarding VRF resistance (*P* = 0.265) and cross-sectional area (*P* = 0.402). The cemented FRC post group showed the lowest resistance to cause VRF (748 ± 239 N), while the cemented Ti post group had the highest VRF resistance (921 ± 328 N).

## Discussion

The data support acceptance of the first null hypothesis, because the results did not show significant differences in vertical root fracture (VRF) resistance. After chewing simulation, none of the roots was completely fractured and the VRF resistance did not differ significantly between adhesively luted fiber-reinforced composite (FRC) or titanium (Ti) posts and their counterparts in the non-adhesive zinc phosphate cement group.

In the present study, the FRC posts were placed with the same cements than the, respectively, compared Ti posts. Whereas the Ti post tested was developed to be used with zinc phosphate and resin cements, FRC posts are recommended to be adhesively luted with resin cements [26]. Zinc phosphate cemented FRC posts were included as control for the potentially reinforcing effects of post type and adhesive luting. Adhesive luting of FRC posts did not significantly increase the VRF resistance, as opposed to the root reinforcement effect of adhesively luted FRC posts reported by other authors [8–12]. On the other hand, consistent with the present fracture load data, it was shown that adhesively luted FRC [15, 16] or Ti [15] posts do not reinforce teeth. Several studies highlighted the challenges of adhesive luting of posts. Factors such as an unfavorable configuration factor, high polymerization shrinkage, interfacial gaps around the post, and difficulties in polymerization inside the post space can negatively influence the adhesive post luting [11, 25, 27]. Therefore, in this study, particular attention was given to the bonding procedure through dentin roughening of the post space, industrial silica and silane coating of the FRC post surface (average roughness *R*_a_ ~ 3 µm) [28], industrial sandblasting with aluminum oxide of the Ti post surface (*R*_a_ ~ 12 µm) [26], and the use of the corresponding dual-curing luting system of the post manufacturer [8, 26]. Surface roughening of both the post and the root dentin has been shown to increase the interfacial bond strength of the luting system by combining chemical bonding with micromechanical retention [26, 28].

Even though no complete root fracture was observed after chewing simulation, there were significant differences in the incidence of dentin defects. The second null hypothesis was, therefore, rejected. Adhesive luting of FRC posts reduced the risk of dentin defects, while zinc phosphate cemented FRC posts caused the highest incidence of vertical cracks. Although adhesive luting of FRC posts is mandatory, variations in bond quality, due, for example, to the bonding procedure, could, therefore, influence the incidence of vertical cracks and future root fractures. In general, vertical cracks in the study caused lower VRF resistance (760 ± 265 N, *n* = 34) than craze lines (936 ± 318 N, *n* = 29). The results are supported by previous stress analyses of FRC post-restored teeth. A recent 3D FEM and fatigue analysis of FRC post-restored teeth showed that adhesive failure of the coronal composite filling increases the dentin stress at the cavity floor, which can lead to VRF [29]. Stress concentrations leading to root fracture were also observed in the corresponding areas of adhesive failure between cement and root dentin [30]. Other 3D FEM analyses of post-restored teeth demonstrated that zinc phosphate cemented or adhesively failed FRC posts generated higher stress in root dentin than metal posts [21, 22]. The latter study also indicated that roots restored with FRC posts are less prone to fracture because the fracture risk of the FRC post and the composite core build-up was higher than that of the root [22]. In the present testing design, the posts were shortened to a level 1 mm below the cutting surface of the root to avoid direct loading. The posts were not evaluated as a retainer for the coronal restoration to assess a possible internal reinforcement of the root and exclude confounding factors such as post and core build-up fracture [24]. Many studies performed fracture resistance measurements and analyses on root canal-treated or post-restored teeth that had been decoronated [6, 7, 15, 24]. From a clinical perspective, however, the results should be interpreted with caution since a coronal restoration/cusp coverage could lead to a more favorable stress distribution towards the root.

The rationale for the simulated periodontal ligament was to avoid external reinforcement of the roots by the surrounding acrylic resin and to be able to easily remove the roots from the acrylic support for microscopic analysis. Fracture resistance of teeth without simulated ligament was higher than those with artificial ligament, as the acrylic resin generated a ferrule effect preventing root fracture [31]. A ~ 250 µm thick rubber coating was chosen to reproduce the damping properties in terms of width and Young’s modulus of the human ligament, consistent with other studies simulating the ligament and using rubbers or similar elastomers around the root [7, 16, 24, 31]. In preliminary tests for the study, the external root surfaces were examined for dentin defects after root canal filling, and no damage was found. An explanation is that possible dentin defects that could be initiated during root canal instrumentation and filling progressed within the inner core dentin adjacent to the root canal wall and were therefore not detectable on the external root surface. Nickel–titanium files with a smaller file taper (4%) were used, as larger file tapers have been shown to reduce the VRF resistance of root canal-filled teeth [6]. In addition, studies showed that single-cone filling techniques like the one used in this study reduce the incidence of dentin defects compared to lateral compaction of gutta percha [5, 6]. The incidence of dentin defects increased with post space preparation as the root canal wall is further reduced and the inner core dentin with a lower Young’s modulus than the more mineralized outer dentin is removed [5, 7, 32].

Regardless of the post group, most dentin defects were observed in the cervical two-thirds of the root samples. One reason might be that the post space was not prepared into the apical third. In addition, the unfilled cervical 1 mm of the post space ensured that the load was transmitted to the dentin walls rather than the post. The loading tended to force the dentin walls outwards, which could cause VRF by wedge effect, as previously described using a similar testing design [33]. Since the posts were not directly loaded, less stress may have been concentrated towards the post tip, resulting in a corresponding stress reduction in the apical root area. The lower incidence of apical defects is consistent with clinical observations in post-restored teeth [34]. Since the tubular density in the root canal decreases from cervical to apical, there are also fewer dentin tubules in the apical part from which cracks can initiate. Morphological examinations on root-filled teeth have provided some evidence that the presence and orientation of dentin tubules influence the crack initiation in root dentin [35]. Cracks were initiated in the peritubular dentin of individual tubules and progressed through the intertubular collagen matrix surrounding the tubules [35]. Fatigue analyses showed that peritubular dentin is more mineralized than intertubular dentin, more brittle and easier to crack [23]. Due to the higher tubule density of the cervical and middle root third, the cracks may have propagated more frequently through the tubules than in the dentin bulk of the apical root third [23].

The prospective power analysis indicated that significant results can be achieved with a sample size of *n* = 18 per group. Although attempts were made to standardize the roots in terms of anatomy using only maxillary lateral incisors, length, cross-sectional dimension, and sample preparation, the coefficient of variation of VRF resistance was between 28% and 36%. This has been reported as a common finding in fracture load testing and to some extent reflects differences in age, dentin microstructure, and morphology between extracted human teeth [24].

## Conclusions

Within the limitations of this study, it can be concluded that the incidence of dentin defects is higher for post-restored roots than for untreated teeth. Adhesively luted FRC posts did not increase the vertical root fracture resistance but reduced the risk of dentin defects. Most dentin defects were craze lines and vertical root cracks.
